# Genetic contributions to stuttering: the current evidence

**DOI:** 10.1002/mgg3.276

**Published:** 2017-02-19

**Authors:** Carlos Frigerio‐Domingues, Dennis Drayna

**Affiliations:** ^1^ National Institute on Deafness and Other Communication DisordersPorter Neuroscience Research CenterNational Institutes of Health

## Abstract

Evidence for genetic factors in persistent developmental stuttering has accumulated over the past four decades, and the genes that underlie this disorder are starting to be identified. The genes identified to date, all point to deficits in intracellular trafficking in this disorder.

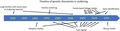

## Introduction

Stuttering is a common speech disorder characterized by word or syllable repetitions or prolongations, and by silent interruptions in the flow of speech known as blocks (Diagnostic and Statistical Manual, 5th Edition, International Classification of Diseases, 11th Edition (2013). Clinical diagnosis typically measures the rates of stuttering dysfluencies while reading or during free speech, and can include the rate of ancillary symptoms, sometimes known as struggle behaviors, during speech (SSI‐4) (Riley 2009). The disorder typically arises in childhood, often as speech and language skills are rapidly developing. While the disorder resolves in the majority of individuals, either spontaneously or with the help of speech therapy, approximately 20–25% of cases do not, leading to a condition known as persistent developmental stuttering, which affects approximately 1% of the general population. Like a number of neurodevelopmental disorders, stuttering affects more males than females (Bloodstein and Ratner 2008). While the underlying causes of stuttering have been speculated upon since antiquity, in the past four decades, evidence for the involvement of genetic factors in this disorder has steadily accumulated. Here, we review the current evidence for heritability of stuttering, genetic linkage studies that have aimed at identifying loci carrying genes causative for this disorder, gene discoveries in stuttering, and functional studies of those genes aimed at illuminating how genetic variants cause this disorder.

### Heritability and modes of inheritance

Twin and adoption studies represent two classical approaches to estimating the heritability of a trait in humans. A total of nine twin studies of stuttering have been published in the past 40 years, involving subjects from widely different linguistic backgrounds (Godai et al. 1976; Howie 1981; Andrews et al. 1991; Felsenfeld et al. 2000; Ooki 2005; Dworzynski et al. 2007; van Beijsterveldt et al. 2010; Fagnani et al. 2011; Rautakoski et al. 2012). These studies have varied greatly in size, ranging from <100 subjects to over 20,000 subjects. They have used different methods of ascertainment and different inclusion criteria, as well as different diagnostic measures and different statistical methods to estimate heritability. As a result, few of these studies are directly comparable and combining data from them, such as for meta‐analyses, has been difficult. However, the results of these studies share several important conclusions. First, monozygotic (MZ) twins consistently display a higher concordance for stuttering than dizygotic (DZ) twins, indicating strong evidence for a genetic component to this disorder. Second, the MZ twin concordance for stuttering is consistently <1, indicating that germ line genetic factors by themselves do not explain all of stuttering. Third, while heritability estimates from these studies have varied, many have produced estimates of high heritability, often exceeding .80 (Table 1).

**Table 1:** Twin studies of stuttering

Study (year)	Language	Number of individuals	Estimated heritability
Godai et al. (1976)	Italian	126	na
Howie (1981)	English	60	na
Andrews et al. (1991)		7620	.71
Felsenfeld et al. (2000)		37	.70
Ooki (2005)	Japanese	3792	.80–.85
Dworzynski et al. (2007)	English	12,892	.60
van Beijsterveldt et al. (2010)	Dutch	21,366	.42
Fagnani et al. (2011)	Danish	22,216	.81–.84
Rautakoski et al. (2012)	Finnish	2289	.82

Adoption studies of stuttering have been more limited, and the first of these was too small to generate statistically significant conclusions (Bloodstein 1961). The second study also did not generate highly statistically significant conclusions, however the authors did fail to find evidence that children adopted by parents who stutter are affected by stuttering at a greater rate than children in the general population (Felsenfeld and Plomin 1997). Although these studies had statistical limitations, they tend to refute the view that children develop stuttering by listening to their parents who stutter.

Several studies have sought to better define the genetic epidemiology of stuttering, and to identify a mode of inheritance in families. Seminal studies with modest size families revealed strong evidence for familial clustering of the disorder (Kidd et al. 1981), and conversely, failed to find evidence that nongenetic factors, such as anxiety or familial attitudes toward speech, contributed to stuttering in families (Cox, Seider, & Kidd, 1984). A few large families with many cases of stuttering have been reported in the historical literature (MacFarlane et al. 1991; Gray 1940). However, these reports did not attempt to apply quantitative methods to test possible modes of inheritance. Other studies have provided inconsistent evidence for a mode of inheritance, with evidence for dominant, recessive, and sex‐modified inheritance reported (Viswanath et al. 2004; Riaz et al. 2005; Wittke‐Thompson et al. 2007). Thus, overall, while the evidence for heritable factors in stuttering is strong, stuttering is most likely a complex genetic trait.

## Linkage and Association Studies

A number of genetic linkage studies have been performed in an effort to identify loci at which causative genes reside. Initial studies produced evidence that was largely suggestive (Shugart et al. 2004; Suresh et al. 2006; Wittke‐Thompson et al. 2007), although greater statistical significance was achieved using conditional models, incorporating factors such as sex‐limited expression or tests of different phenotypic classifications. The linkage loci identified in these early studies showed little if any overlap (Table 2), and results of gene finding studies at these loci have not been reported.

**Table 2:** Linkage studies of stuttering

Study (year)	Chromosome region	Statistical strength level
Shugart et al. (2004)	18p	Suggestive
	18q	Suggestive
Riaz et al. (2005)	12q	Highly significant
Suresh et al. (2006)	2q	Significant
	7q	Suggestive
	9p	Suggestive
	12q	Suggestive
	13q	Suggestive
	15q	Suggestive
	20p	Suggestive
	21q	Highly significant
Wittke‐Thompson et al. (2007)	3q	Suggestive
	13q	Suggestive
	15q	Suggestive
	9q	Significant
	3q	Significant
	13q	Significant
	2q	Suggestive
	5q	Significant
Raza et al. (2010)	3q	Highly significant
Raza et al. (2012)	16q	Highly significant
Raza et al. (2013)	2p	Highly significant
	3p	Significant
	3q	Highly significant
	14q	Highly significant
	15q	Highly significant
Domingues et al. (2014)	10q	Significant

One population‐based genome‐wide association study (GWAS) of stuttering was presented in a PhD thesis by Kraft (2010). The sample size of this study was modest, and no associations were identified that approached the currently accepted significance criteria for association of *P *=* *5 × 10^−8^. While this study was of limited statistical power, the results were consistent with the view that variants in no single gene serve as a major cause of stuttering in the overall population.

Subsequent linkage studies then focused on families with mating patterns not commonly found in the United States. These produced more definitive evidence for linkage, with loci on chromosomes 3, 12, and 16 identified in highly consanguineous families from Pakistan (Riaz et al. 2005; Raza et al. 2010, 2012) and on chromosomes 2, 3, 14, and 15 in a large polygamous family from Cameroon, West Africa (Raza et al. 2013) (Table 2). These linkage scores substantially exceeded criteria for genome‐wide significance, and two of these linkage signals have enabled subsequent gene identification at those loci.

## Gene Identification

### *GNPTAB*

The first gene suggested to play a causative role in stuttering was initially found on the basis of a robust linkage signal on chromosome 12q observed in a sample of 44 consanguineous families from Pakistan (Riaz et al. 2005). Sequencing the genes at this locus led to the discovery of a nonsynonymous coding variant in the *GNPTAB* gene, which encodes the enzyme N‐acetylglucosamine‐1‐phosphotransferase, (hereafter, the “phosphotransferase”), (OMIM#607840, GenBank ID 79158). In this mutation, the highly conserved glutamic acid at amino acid position 1200 was replaced by lysine (p.Glu1200Lys, c.G3598A). This variant was not found in a cohort of 96 normal Pakistanis, nor in any human sequence data base at that time (Kang et al. 2010). However, it was found in the affected members of four of the 44 Pakistani families originally included in the genome‐wide linkage search. This suggested that the *GNPTAB* p.Glu1200Lys mutation, which was subsequently shown to be a founder mutation (Fedyna et al. 2011), underlies the disorder in ~ 9% of Pakistani families with multiple cases of persistent stuttering. Subsequent investigations revealed the presence of other rare mutations in *GNPTAB* in families and unrelated individuals with persistent stuttering from Pakistan, North America, England, and Brazil (Raza et al. 2015a). A significant difference in the rate of putative mutations in this gene (defined as rare coding variants at conserved sites) was confirmed in North American and Pakistani cases compared to population‐matched controls. All the putative mutations identified in this gene in cases and controls were heterozygous missense amino acid substitutions.

### *GNPTG*,* NAGPA,* and the lysosomal targeting pathway

The finding of mutations in the *GNPTAB* gene in stuttering motivated additional studies of two functionally related genes. *GNPTG* (OMIM#607838, GenBank ID 84572) encodes a protein subunit that combines with the product of the *GNPTAB* gene to form the functional phosphotransferase enzyme, while the *NAGPA* gene (OMIM#607985, GenBank ID 51172) encodes the enzyme N‐acetylglucosamine‐1‐phosphodiester alpha‐N‐acetylglucosaminidase, also referred to as the uncovering enzyme (UCE). These two enzymes comprise a simple two‐step biochemical pathway, which serves to attach a mannose‐6‐phosphate moiety that acts as signal that targets a diverse group of hydrolytic enzymes to the lysosome (Kornfeld 1987) (see Gene Function and Medical Genetics, below). An excess of putative mutations in *GNPTG* and *NAGPA* were found in stuttering cases compared to population‐matched unaffected controls and public databases of individuals largely unphenotyped for stutttering (Kang et al. 2010; Raza et al. 2015a). As in *GNPTAB*, the variants in these two genes observed in cases were almost exclusively missense amino acid substitutions. It is worth noting that *GNPTG* and *NAGPA* lie close to each other on chromosome 16p1.3, but to date no family‐based linkage study has produced a significant signal at this location.

### *AP4E1*

Studies of a large Cameroonian family produced evidence for multiple loci linked to stuttering, with loci on chromosomes 2, 3, 14, and 15 each showing linkage in different branches of the pedigree (Raza et al. 2013). Two mutations in cis on the same haplotype (p.Val517Ile (c.G1549A) and p.Glu801Lys (c.G2401A)) in the *AP4E1* gene, encoding adaptor protein complex 4, epsilon 1 subunit (OMIM#607244, GenBank ID 23431) cosegregated with each other and with stuttering in the branch of this family showing linkage to chromosome 15. These two mutations were absent from 96 Cameroonian controls and from >7000 individuals in public databases, but were found in two of 96 unrelated Cameroonian individuals who stutter. Other mutations in this gene were identified in affected individuals from North American, South American, European, African, and South Asian populations. The rate of putative mutations in this gene was significantly higher in North American, Cameroonian, and Pakistani cases compared to matched control populations. The rate of variants in Brazilian cases was not significantly higher than in Brazilian controls but overall, the rate of putative mutations was substantially higher in all case groups compared to similar populations in databases (Raza et al. 2015b). In addition, the types of putative mutations found in cases were different than those in controls. Stuttering cases had many predicted loss‐of‐function variants in *AP4E1*, including deletions, frameshifts, nonsense, and splice site variants, while only missense substitutions were observed in controls. All mutations in cases and controls were present in a single copy. Additional evidence for a causative role of *AP4E1* mutations in stuttering came from functional studies described below.

### Contributions to stuttering

Estimates of the contribution of mutations in these four genes to the total cases of stuttering are limited by several factors. These factors include what classes of stuttering are included (all cases, persistent, recovered), the definition of a likely causative mutation, the ancestry of the populations, and the sample sizes. In one recent estimate, putative causative mutations in the *GNPTAB* gene were found in 87 of 1013 unrelated cases (8.6%) (Raza et al. 2015a). This study also found putative mutations in *GNPTG* in 45 of these 1013 cases (4.4%), and putative mutations in *NAGPA* in 32 of these 1013 cases (3.2%). Putative causative mutations in *AP4E1* were found in 34 of 936 unrelated cases (3.6%) (Raza et al. 2015a,b).

Taken together, these results could suggest that a mutation in these four genes cumulatively account for approximately 20% of unrelated cases of persistent stuttering. However, this figure is tempered by the observation of other rare nonsynonymous coding variants in 8% of subjects of matched ethnicity in population databases. This indicates that rare coding variants in these four genes exist in a nontrivial fraction of the general population. Thus, the amount of stuttering attributable to mutations in these genes might better be described as the increase in rate observed in cases (20%) over that observed in controls (8%), suggesting that 12% of cases are due to contributions from mutations in these four genes. However, individuals in public databases are not phenotyped with respect to speech or speech history. Persistent stuttering affects approximately one percent of the population, and childhood developmental stuttering affects approximately 4–5 percent of the population (Bloodstein and Ratner 2008). Thus, some of the individuals in these databases are likely to be current or former stutterers. If so, the estimate of the contribution of these four genes might be closer to 20%.

A caution worth noting is that while the stuttering case and control groups described above have been closely matched geographically, they have not been carefully matched genetically. High‐resolution genome‐wide genetic data would allow more detailed comparison of cases with controls, and could rule out subtle ancestry differences in cases and controls that could affect previous conclusions.

## Gene Function and Medical Genetics

*GNPTAB* and *GNPTG* are well known in medical genetics as the site of mutations that cause Mucolipidosis Types II (MLII) and III_A_ (MLIII_A_). MLII and MLIII_A_ are rare, fatal, recessive lysosomal storage disorders that are due to a failure of the mechanism that targets ~50 acid hydrolases to their proper location in the lysosome (Lee et al. 2011). This targeting is accomplished by the addition of a mannose‐6‐phosphate to N‐linked oligosaccharides on these enzymes. The mannose‐6‐phosphate targeting signal is recognized by one or more mannose‐6‐phosphate receptors that function to route such enzymes to the lysosome (Lee et al. 2011). The addition of the mannose‐6‐phosphate to these enzymes takes place via the two‐step enzymatic pathway described above (Lee et al. 2011).

Mutations in *GNPTAB* were originally found in MLII, a disorder that is diagnosed at birth, has severe symptoms, and is associated with very low phosphotransferase activity in vitro (0–3% of normal). Complete loss‐of‐function mutations are often found in this disorder, and affected individuals die within the first decade of life. MLIII_A_ is less severe, with affected individuals living into adolescence and young adulthood. Mutations in *GNPTG* have been more often found in MLIII_A_, which in addition to milder symptoms, can be associated with slightly higher phosphotransferase activity (3–15%) in vitro. All MLII and MLIII_A_ patients studied to date are homozygous for mutations in *GNPTAB* or *GNPTG*, consistent with Mendelian recessive transmission of these disorders. Interestingly, mutations in *NAGPA* have not been found in mucolipidosis or any other human disorder other than stuttering.

Early studies of unrelated stuttering individuals carrying mutations in *GNPTAB* and *NAGPA* found that almost all subjects carried a single copy of the mutant allele, and that these subjects displayed no signs or symptoms associated with MLII or MLIII_A_, nor any evidence of more general neurologic impairment (Kang and Drayna 2012). One limited in vitro study looked at the effect of several stuttering mutations found in *NAGPA*, and found these variants were associated with an approximately 50% decrease in activity (Lee et al. 2011). In addition, the mutations in *GNPTAB* and *GNPTG* found in stuttering have been almost all missense mutations, unlike complete loss‐of‐function mutations frequently seen in mucolipidosis, and there is very little overlap in the location of mutations in these genes found in stuttering and those found in mucolipidosis (Raza et al. 2015a). The differences between mutations in these genes found in mucolipidosis and those found in stuttering are summarized in Table 3.

**Table 3:** Overview of mutations in *GNPTAB*,* GNPTG*, and *NAGPA* found in stuttering versus those found in mucolipidosis

*GNPTAB*,* GNPTG*, and *NAGPA* mutations	
Stuttering	Mucolipidosis
Typically heterozygous	Homozygous
Typically missense	Many null alleles
Modest reduction of enzymatic function	Very low to zero enzymatic function
Different sites than mutations in mucolipidosis	Different sites than mutations in stuttering

### *AP4E1*

The *AP4E1* gene encodes the epsilon subunit of the Adaptor Protein 4 (AP4) complex. To date, there have been five such complexes described in humans, designated AP1‐AP5. These complexes each contain four subunits, typically an alpha and a beta subunit plus gamma, delta, epsilon, or mu subunits (Dell'Angelica et al. 1999; Hirst et al. 1999). They serve as adaptors that recognize cargo contained within endosomes, and they participate in intracellular traffic sorting to deliver endosomal cargoes to their correct location within the cell (Matsuda et al. 2008; Burgos et al. 2010). Mutations found in stuttering cases can serve to disrupt the assembly of the AP4 complex (Raza et al. 2015b). While no in vitro assay of AP4 function currently exists, an important clue to the normal function of this complex came from the recognition that the NAGPA protein contains an AP4 recognition sequence near its carboxy‐terminus. Yeast 2‐hybrid studies showed that AP4 components physically interact with NAGPA, and that mutations of the amino acids in the predicted AP4 recognition sequence in the carboxy‐terminal region of NAGPA abolish these interactions (Raza et al. 2015b). Thus, it appears that AP4E1 has a close functional relationship with the lysosomal targeting machinery.

Literature reports of mutations in *AP4E1* have been few (Abou Jamra et al. 2011; Moreno‐De‐Luca et al. 2011; Kong et al. 2013). Patients with recessive loss‐of‐function mutations in this gene typically display many neurologic deficits, including spastic paraplegia, cerebral palsy, and developmental delay, and may also have skeletal abnormalities. While significant speech deficits have been described in some of these subjects, it is not clear if these are a primary manifestation of AP4E1 deficiency, or if they are secondary to the more general neurodevelopmental impairments in these patients. None of the mutations reported in these patients have been found in individuals with stuttering. *AP4E1* mutations in stuttering subjects are all heterozygous, although they include loss‐of‐function variants such as deletions, frameshifts, stop gain mutations, and splice site mutations. The few stuttering subjects who carry such mutations and have been examined clinically have displayed no neurologic or other clinical deficits other than stuttering.

## Current Challenges and Future Prospects

The genes identified in stuttering to date, all point to a single process, intracellular trafficking, as the site of the cellular defect in this disorder. Intracellular trafficking deficits are an emerging concept in neurologic disorders (Neefjes and van der Kant 2014). Although such deficits were originally recognized in rare Mendelian disorders, they are now suggested to be important in the genesis of common or genetically complex disorders such as Parkinson's Disease (MacLeod et al. 2013), Alzheimer Disease (Cataldo et al. 2000) and Huntington Disease (Caviston and Holzbaur 2009). However, the four genes found to date, at most, account for only 20% of persistent stuttering cases, leaving most of such cases currently unexplained. It is clear that more genes remain to be identified, in particular, at loci where highly significant linkage scores have been reported, which include chromosomes 2, 3p, 3q, 10, 14, and 16. The fact that these genes have not yet been found despite strong linkage scores suggests that, while mutant alleles with large effect can exist for stuttering, identifying these causative genes will remain challenging. Although the genes identified to date are all functionally related, the nature of the genes remaining to be identified is unknown, and these genes could lead us to other, perhaps unanticipated pathologic mechanisms in stuttering. In addition, twin studies tell us that some cases of stuttering are likely due to noninherited causes. Thus, the identification of all the genes that contribute to stuttering will still leave us with an incomplete picture of the genesis of this disorder.

Meanwhile, the four genes and presumptive cellular mechanism identified to date open a number of possible paths to a better understanding of the neuropathology of stuttering. Stuttering subjects who carry a mutation in one of the four currently identified genes have no identifiable neurological deficits other than stuttering. As a result, we have hypothesized that the neuropathology caused by these mutations is limited to a small and specialized population of neurons. Efforts to identify such neurons would be greatly aided by a genetically tractable animal model.

Adult and newborn mice are highly vocal (Ehret 1976; Maggio and Whitney 1985; Romand and Ehret 1990; Stowers et al. 2002; Liu et al. 2003; Gourbal et al. 2004; Panksepp et al. 2007) and could perhaps provide a model of some aspects of human speech. A demonstration of the validity of the mouse as such a model would allow the extensive genetic tools available in the mouse to be applied to studies of speech disorders. Mouse vocalizations are frequently above 20 kHz in frequency and thus in the ultrasonic range for humans. This requires specialized equipment and software for recording and analysis of these vocalizations (Brudzynski et al. 1999; Holy and Guo 2005; Portfors 2007). These ultrasonic vocalizations are under substantial genetic control (Kikusui et al. 2011), and while a number of features of mouse vocalization are unsuitable as a model of human speech, it was hypothesized that they may be useful as a model for the volitional control of vocalization, deficits in which represent an important feature of human stuttering.

Mice were engineered to carry a Gnptab p.Glu1176Lys mutation, the mouse homolog of the human GNPTAB p.Glu1200Lys mutation found in Pakistani stuttering families and unrelated cases. Mice homozygous for this mutation display no obvious abnormalities. They grow and breed normally, and produce litters of normal size and sex distribution. No abnormalities are observed by hematoxylin and eosin (H&E) staining of tissue sections. They also exhibit normal behavior on a wide range of standard laboratory behavioral tests. In addition, many aspects of their ultrasonic vocalizations were indistinguishable from their wild‐type littermates. However, a consistent and statistically significant difference was observed in the timing of their vocalizations, with homozygous mutant animals displaying less vocalization due to longer pauses in their vocalizations compared to their wild‐type littermates. Applying the same analytical method to the speech of humans who stutter and carry mutations in the *GNPTAB* gene demonstrated a similar vocalization phenotype. Thus, mice carrying human stuttering mutations can replicate an important clinical feature of the disorder (Barnes et al. 2016). This suggests that the rich array of genetic and other experimental tools available in the mouse, which include the ability to express mutations in specific cell types and at specific times in development, can be exploited to better define the neuropathology that underlies persistent stuttering.

Finally, next‐generation DNA sequencing technologies represent the new standard in human genetic and genomic analyses. While linkage, population‐based association, and gene identification studies in stuttering have made gradual progress, the ability to assay all the genetic variation in individual exomes and genomes presents improved prospects for identifying more of the genes that underlie stuttering. Knowledge of how these gene defects can, individually or acting together, lead to the observed pathology provides the prospect for improving our understanding and treatment of this long‐enigmatic disorder.
